# Coatings for FEL optics: preparation and characterization of B_4_C and Pt

**DOI:** 10.1107/S1600577517016095

**Published:** 2018-01-01

**Authors:** Michael Störmer, Frank Siewert, Christian Horstmann, Jana Buchheim, Grzegorz Gwalt

**Affiliations:** a Institute of Materials Research, Helmholtz-Zentrum Geesthacht, Max-Planck-Strasse 1, D-21502 Geesthacht, Germany; b Helmholtz-Zentrum Berlin, Albert-Einstein-Strasse 15, 12489 Berlin, Germany

**Keywords:** FEL, X-ray mirrors, FEL optics, coatings

## Abstract

Large X-ray mirrors are required for beam transport at both present-day and future free-electron lasers (FELs) and synchrotron sources worldwide. The demand for large mirrors with lengths up to 1 m single layers consisting of light or heavy elements has increased during the last few decades. Accordingly, surface finishing technology is now able to produce large substrate lengths with micro-roughness on the sub-nanometer scale. At the Helmholtz-Zentrum Geesthacht (HZG), a 4.5 m-long sputtering facility enables us to deposit a desired single-layer material some tens of nanometers thick. For the European XFEL project, the shape error should be less than 2 nm over the whole 1 m X-ray mirror length to ensure the safe and efficient delivery of X-ray beams to the scientific instruments. The challenge is to achieve thin-film deposition on silicon substrates, benders and gratings without any change in mirror shape. Thin films of boron carbide and platinum with a thickness in the range 30–100 nm were manufactured using the HZG sputtering facility. This setup is able to cover areas of up to 1500 mm × 120 mm in one step using rectangular sputtering sources. The coatings produced were characterized using various thin-film methods. It was possible to improve the coating process to achieve a very high uniformity of the layer thickness. The movement of the substrate in front of the sputtering source has been optimized. A variation in B_4_C layer thickness below 1 nm (peak-to-valley) was achieved at a mean thickness of 51.8 nm over a deposition length of 1.5 m. In the case of Pt, reflectometry and micro-roughness measurements were performed. The uniformity in layer thickness was about 1 nm (peak-to-valley). The micro-roughness of the Pt layers showed no significant change in the coated state for layer thicknesses of 32 nm and 102 nm compared with the uncoated substrate state. The experimental results achieved will be discussed with regard to current restrictions and future developments.

## Abstract 2

Boron carbide and platinum are two suitable coating materials for X-ray mirrors at free-electron lasers worldwide. The achieved thickness uniformity for boron carbide is less than 1 nm peak-to-valley over 1500 mm mirror length.

## 1. Introduction   

Long X-ray mirrors are required for photon beam transport and shaping in the beamlines at free-electron lasers (FELs) and synchrotron sources. Surface finishing and coating technology of such mirrors is challenging, since the technical specifications are extremely tight (US DOE, 2013 ▸). There are VUV and hard X-ray FEL sources in operation such as FLASH (Germany) (Tiedtke *et al.*, 2009 ▸), LCLS at SLAC (USA) (Boutet & Williams, 2010 ▸), FERMI at ELETTRA (Italy) (Cocco *et al.*, 2010 ▸), SACLA (Japan) (Ishikawa *et al.*, 2012 ▸) and SwissFEL at PSI (Switzerland) (Patterson *et al.*, 2010 ▸). The European XFEL project (Altarelli, 2011 ▸) has now entered the operation phase. The European XFEL will provide new research opportunities to users from science domains as diverse as physics, chemistry, geosciences, materials science and biology (Tschenscher *et al.*, 2017 ▸). A continuous improvement of measurement tools is needed as well (Siewert *et al.*, 2014 ▸; Siewert, 2013 ▸). For the European XFEL project, a radius of curvature of more than 6000 km is required, and needs to be measured (Sinn *et al.*, 2011 ▸). The required technical specifications lead to a shape error of less than 2 nm peak-to-valley (PV) for the mirrors. These extraordinarily high specifications are needed to preserve the wavefront and maintain the coherence of the FEL beam. The specifications on polishing errors are about a factor of ten more stringent compared with well established mirrors for synchrotron radiation (European XFEL, 2016 ▸). The first FEL coatings with amorphous boron carbide (B_4_C) on single-crystalline silicon substrates have been deposited and investigated (Störmer *et al.*, 2016*a* ▸). The Helmholtz-Zentrum Geesthacht (HZG) sputtering facility was used to coat the worldwide unique 1 m-long silicon substrates to extraordinarily high specifications. The achieved coating properties demonstrated that the PV shape error was retained below 2 nm along the optical aperture of about 1 m length. Several B_4_C coatings were manufactured to characterize the precision, stability and repeatability of the coating process and their properties. A particular scientific instrument at the European XFEL, the Materials Imaging and Dynamics (MID) beamline, requires mirrors with a second stripe of coating materials, which possess a higher critical angle than the standard coating of B_4_C (Madsen *et al.*, 2013 ▸). The operation range should be increased to higher angles at 25 keV using a second metallic coating stripe with a very high-*Z* element like platinum (Pt). Lateral control of the thickness is also very important for multilayers with and without lateral gradients (Störmer *et al.*, 2016*b* ▸).

This article presents our latest coating developments relating to FEL coatings with low-*Z* B_4_C and high-*Z* Pt. The challenge is to deposit stable and uniform coatings over a long deposition length. The first part is primarily dedicated to B_4_C coatings and their newest experimental results due to thickness uniformity along the whole deposition length of 1500 mm, which were improved distinctly compared with former results (Störmer *et al.*, 2016*a* ▸). In the second part the X-ray optical properties of Pt coatings are introduced according to layer thickness, density and micro-roughness. Finally, the layer thickness values in the tangential direction of the mirrors along 1500 mm are shown for the most important FEL coatings.

## 2. Experimental   

Boron carbide and platinum coatings were deposited on silicon substrates using the 4.5 m-long HZG magnetron sputtering facility. The maximum deposition length is 1500 mm. The ultra-high-vacuum chamber is evacuated by a turbo-molecular pump and a cryo-pump. A laminar-flow box is used for the pretreatment and cleaning of the substrates. Two super-polished silicon substrates (50 mm × 20 mm × 10 mm; Gooch & Housego) with a very low surface roughness of less than 0.1 nm r.m.s. (specified by the manufacturer and measured in the high-spatial-frequency range) were coated with different thicknesses of Pt. Typical parameters of the deposition chamber are a base pressure of less than 1 × 10^−7^ hPa, a source-to-substrate distance of about 13 cm and an argon gas pressure of 0.13 Pa. The generator power values were 800 W MF (mid-frequency) for B_4_C at a rectangular source (304.8 mm × 88.9 mm) and 80 W DC for Pt at a circular source (diameter of 76.2 mm). Small silicon substrates (20 mm × 60 mm × 0.7 mm) for test coatings were fixed on a large mirror dummy as described previously (Störmer *et al.*, 2016*a* ▸). In the case of B_4_C we used the maximum deposition length of 1500 mm; for Pt we reduced the process to 500 mm in order to save cost and material. All coatings were investigated by means of X-ray reflectometry using Cu radiation (8048 eV) with parallel beams due to the use of Göbel mirrors (Schuster & Göbel, 1995 ▸; Schuster *et al.*, 1999 ▸). A D8 Advance (Bruker AXS) diffractometer with reflectometry stage and knife edge was employed to investigate thin films on test and super-polished silicon substrates. For the reflectometry and rocking scans shown below, very precise goniometers with a precision below ±0.0005° were used, which are commercially available. The reflectometry scans were recorded with a step width of 0.003°. The precision and limits of reflectometry measurements have been described in more detail elsewhere (Holy *et al.*, 1999 ▸). The reflectivity scans were analysed using the simulation software package *LEPTOS R* (Bruker AXS) and *IMD* software (Windt, 1998 ▸), which allow the modeling of all important parameters such as thickness, density and roughness. It is worth mentioning that the limited resolution of the instrumentation is taken into account. Typically, the accuracy of the layer thickness determination is some 0.01 nm. Densities can be determined down to 1%. Hereby, a good density contrast between film and substrate would be helpful. The optical constants used were taken from the Henke tables (Henke *et al.*, 1993 ▸). Micro-roughness investigations were carried out using white-light interferometry at the Helmholtz-Zentrum Berlin. The surface analysis was used to measure the micro-roughness of various platinum coatings on super-polished substrates. A white-light interferometer (Micromap Promap 512) was used with Mirau-type interferometer objectives studying the micro-roughness in the mid- and high-spatial-wavelength range using different magnifications.

## 3. Results and discussion of FEL coatings   

The experimental results of FEL coatings are divided into three parts and the results are discussed according to the technical requirements for X-ray mirrors. The first part presents the current status in the field of B_4_C coatings. The second part is dedicated to the X-ray optical properties of platinum coatings. Platinum is interesting as a high-*Z* material for total-reflection coatings at certain photon energies. Micro-roughness results are shown in the third part. Finally, the thickness uniformity of various FEL coatings is presented and discussed.

### 3.1. Reflectivity scans and thickness uniformity of B_4_C coatings   

**Figure 1:**
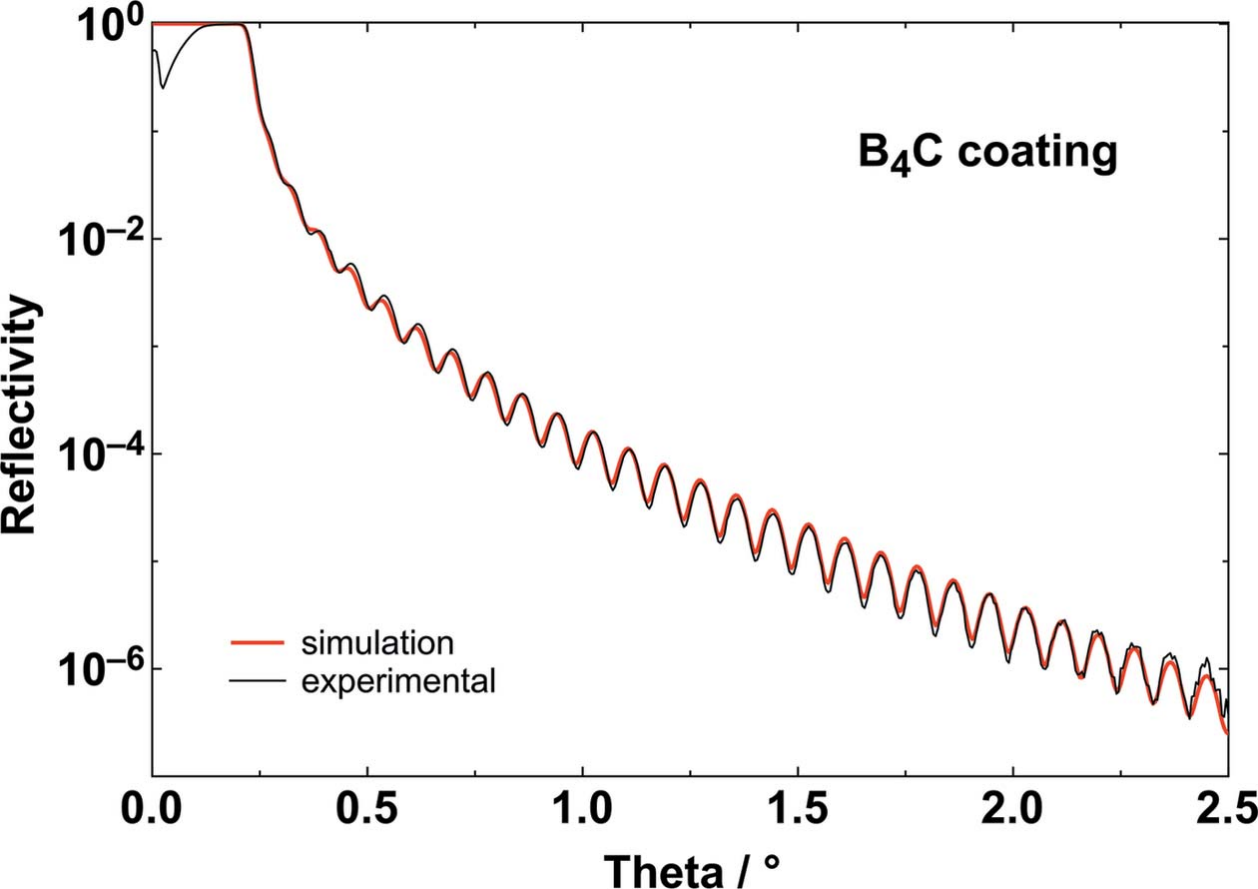
Reflectometry measurement and simulation of a B_4_C coating.

Fig. 1 ▸ shows the measured and simulated X-ray reflectivity scans of a B_4_C coating as a function of incidence angle. As expected for a single-layer system (Atwood, 1999 ▸), Kiessig fringes are clearly visible due to interference of the reflected X-rays at both interfaces of the single layer, *i.e.* film/air and film/substrate. For the simulation it was important to take into account that silicon naturally exhibits a top layer of silicon oxide. The thickness values used for the simulated two-layer system are 51.4 nm and 1.0 nm for B_4_C and SiO_2_, respectively. The roughness values are about 0.5 nm, which are appropriate for a calibration sample. The critical angle is at 0.22°, *i.e.* 3.8 mrad, which means that the film density of the magnetron-sputtered B_4_C layer is 2.37 g cm^−3^ and this is 94% from the bulk value of 2.52 g cm^−3^ (Thévenot, 1990 ▸). Previous results are in good agreement (Störmer *et al.*, 2010 ▸, 2011 ▸, 2016*a* ▸; Kozhevnikov *et al.*, 2015 ▸).

**Figure 2:**
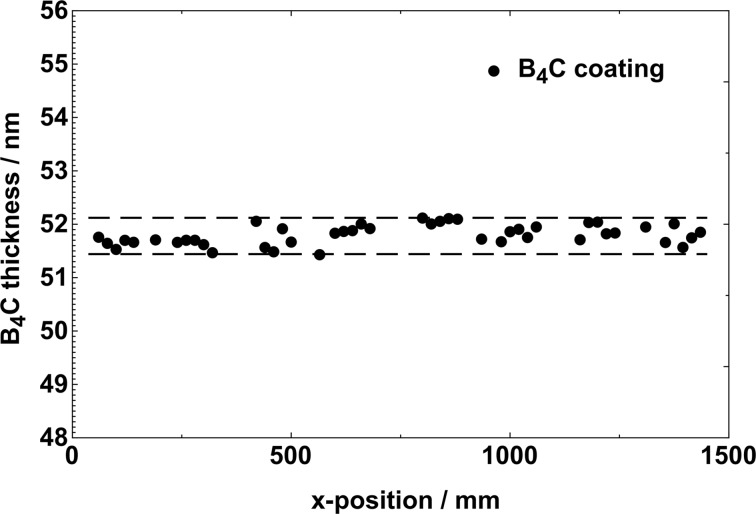
Thickness values of a B_4_C coating at 44 positions along the *x* position (*i.e.* the tangential direction) of a 1.5 m-long mirror. The mean value amounts to 51.8 nm. The PV value is 0.69 nm.

The specular reflectivity scans of multiple B_4_C coatings were measured at 44 positions on 12 Si test substrates (each 120 mm × 30 mm), which were placed along the center of a mirror dummy (the *x* direction). A substrate width of 30 mm is fully sufficient, because the final mirror is coated with a 20 mm-wide B_4_C stripe on the optical area. Four important aspects were necessary to achieve excellent conditions to ensure a uniform coating process: (1) Rectangular sputtering sources were used to attain a uniform deposition profile. (2) A waisted mask in front of the sputtering target improved the deposition further due to reduced coating in the central area. (3) The inclination of the substrate on the carrier has to be controlled and adjusted precisely with respect to the vertical axis of the mirror dummy. (4) Finally, the carrier movement has been optimized so that the distance between source and mirror is constant during the whole travel of the mirror from the beginning to the end of the coating process. When the positioning and movement of the mirror has finished, an excellent thickness uniformity of the B_4_C layers can be achieved with 0.7 nm PV along 1.5 m, which, to our knowledge, is the best value ever reported. In Fig. 2 ▸ all measured B_4_C layer thicknesses over a 1500 mm mirror length are plotted. The mean, minimum and maximum values are 51.8 nm, 51.4 nm and 52.1 nm, respectively. Below, the thickness uniformity of B_4_C coatings that was obtained will be compared with the experimental results for platinum, tungsten and carbon coatings.

### 3.2. Reflectivity scans and thickness uniformity of Pt coatings   

**Figure 3:**
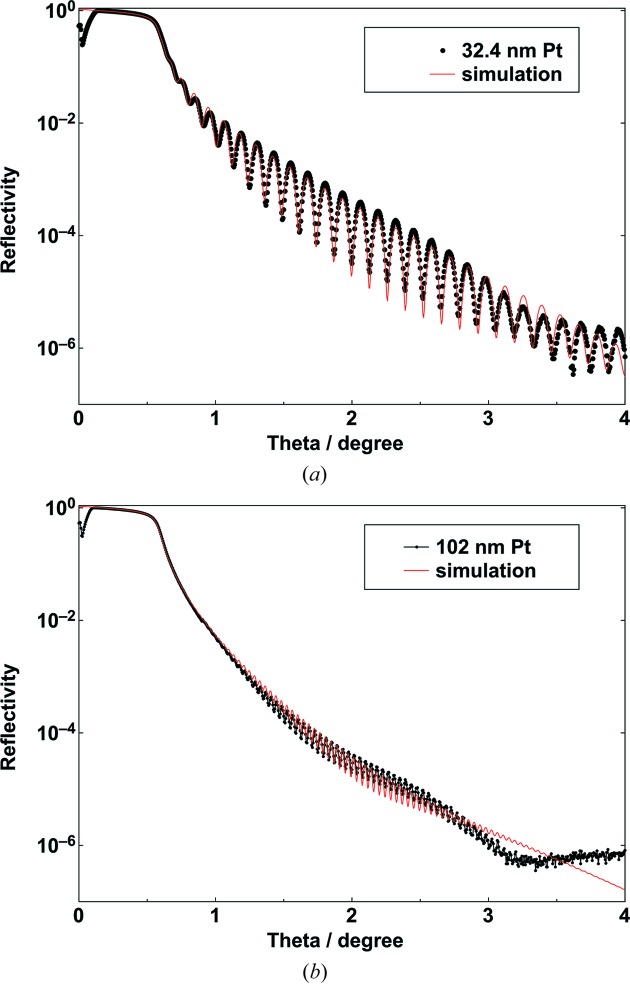
Specular X-ray reflectivity as a function of the incidence angle for two Pt coatings with thicknesses of 32.4 nm (*a*) and 102 nm (*b*). The scans were performed using Cu radiation (8048 eV).

A Pt coating, as a high-*Z* stripe, is very interesting for increasing the critical angle for total external reflection at 25 keV, as mentioned above. Therefore, we coated two Pt coatings in order to characterize the X-ray optical properties such as film thickness, density and roughness. Very high precision substrates with a very low roughness of less than 0.1 nm r.m.s. were used for this important quality check. It is often expected that the roughness of a metal layer will increase with thickness. In the early stages of film growth, metal films deposited on oxide substrates form islands (Ohring, 2002 ▸). In the later stages, surface roughness evolves and remains even after percolation of the islands. Once the deposition conditions have been carefully chosen, then the roughness evolution can be reduced and sometimes even improved. Some micro-roughness measurements, obtained using white-light microscopy, are shown below. In Figs. 3(*a*) and 3(*b*) ▸, the measured and simulated specular reflectivity scans for two different thicknesses are shown over seven orders. The X-rays penetrate the high-*Z* material Pt at higher angles compared with the low-*Z* material B_4_C. The critical angle at 8 keV (Cu radiation) for the Pt is 0.56°, which means that the film density of the magnetron-sputtered Pt layer is about 20 g cm^−3^ and 95% of the bulk value. In the first scan, 27 Kiessig oscillations are clearly visible for the 32 nm-thick Pt layer due to constructive interference. The frequency of the fringes is clearly higher for the 102 nm-thick Pt layer. The amplitude is reduced due to increased absorption in the thicker layer. Above an incidence angle of 3°, both scans are constricted and a slight difference in the decay is measured. This can be explained by a very thin top layer of platinum oxide with a thickness of about 1 nm. It is planned to perform some analysis using X-ray photoelectron spectroscopy for clarification. It is worthwhile noting that some 12 year old Pt films have been analysed with X-ray reflectometry; however, the reflectivity scans remained unchanged (not shown here). There is no evidence for any degradation of Pt films in common laboratory atmosphere.

**Figure 4:**
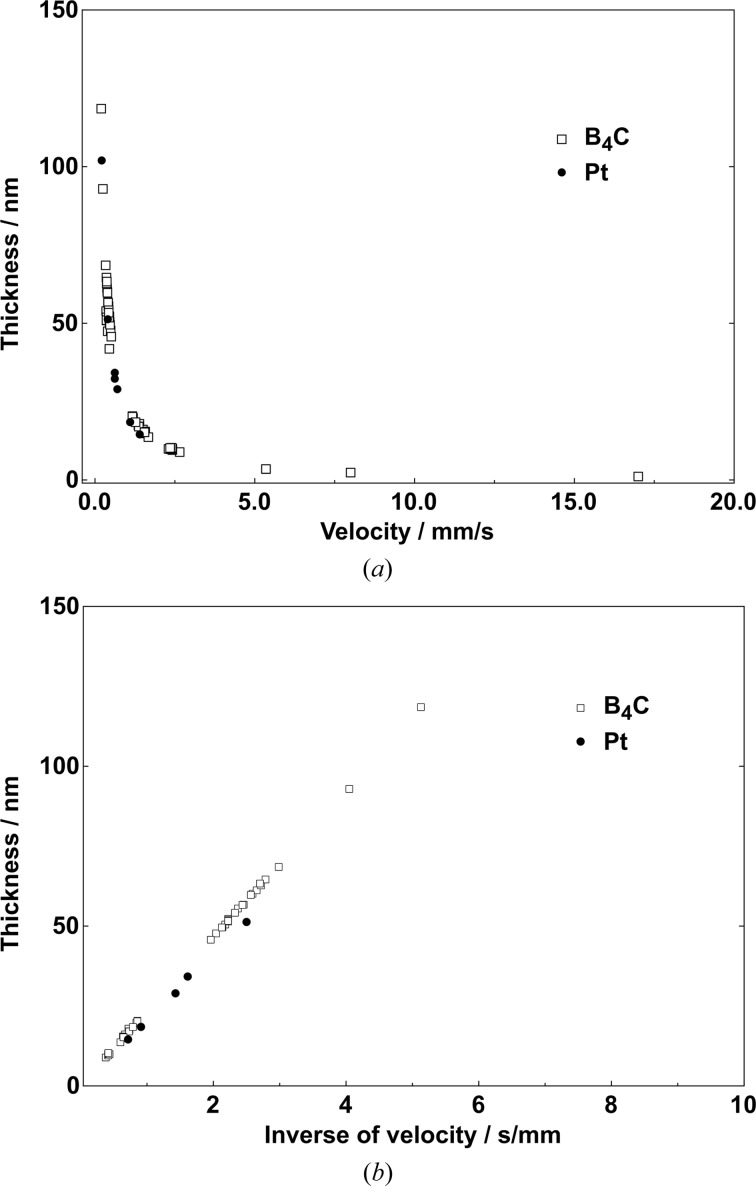
Layer thickness values of magnetron-sputtered platinum and boron carbide as a function of the carrier velocity during deposition (*a*) and of the inverse of the velocity (*b*). The latter is used as a straight line for calibration.

A set of calibration samples were coated to determine the dependence of carrier velocity during Pt deposition and layer thickness (see in Fig. 4*a* ▸). Experimental results from B_4_C deposition are shown as well. An inverse dependency is expected since a high velocity means that the time in front of the sputtering source is short resulting in a thin layer; alternatively a low velocity results in a thick layer. Some extreme examples of B_4_C coatings should be mentioned here, *e.g.* a velocity of 17 mm s^−1^ leads to a thickness of 1 nm, and 0.2 mm s^−1^ results in a 0.12 µm thickness. In Fig. 4(*b*) ▸ the reciprocal of the velocity has been plotted as a function of thickness. By applying a linear fit to the data, it is possible to determine the velocity for a required thickness. Our experimental experience has shown that this method ensures a very high precision to obtain a desired layer thickness. The data correlation coefficients are 0.9992 and 0.9999 for the Pt and B_4_C materials, respectively. It is important to mention that the slope of the fitted straight line depends on the sputtering conditions such as Ar gas pressure, type of gas, sputtering material, target–substrate distance and generator power.

**Figure 5:**
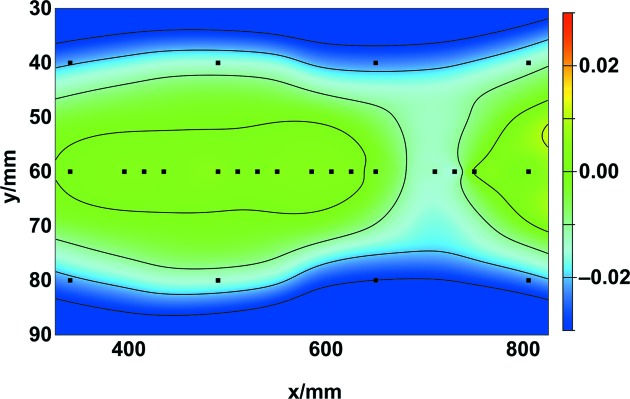
Deviation of the local thickness values normalized by the mean value of a Pt coating. The thickness values are determined at 24 positions (squares) over an optical area of 500 mm by 60 mm. The mean value amounts to 34.2 nm (in the center). The PV value is 1.04 nm over the whole aperture, which is approximately 3%. The colored *z*-scale is the normalized layer thickness difference given by (*t* − *t*_m_)/*t*_m_, where *t*_m_ is the mean thickness and *t* is the measured local thickness. Contour lines were interpolated in order to show the changes. The values of normalized difference slightly decrease towards the outer regions of the optical area.

The thickness uniformity of the Pt layers is characterized across an optical area of 500 mm × 60 mm (Fig. 5 ▸); such dimensions are characteristic of a medium-sized mirror for FEL applications. The Pt layer thicknesses were determined at 24 positions over the whole area, of which 16 values were along the center (the *x* direction or the tangential direction of the mirror). The mean, minimum and maximum thickness values were 34.0 nm, 33.2 nm and 34.2 nm, respectively. The PV value was about 1.0 nm. The uniformity of the Pt layers could be improved by using a mask in front of the 3-inch sputtering target. Another way to improve the uniformity would be to work with a larger rectangular sputtering target. Unfortunately, this is not feasible due to the high price of such a large Pt target.

### 3.3. Micro-roughness analysis of Pt coatings   

**Figure 6:**
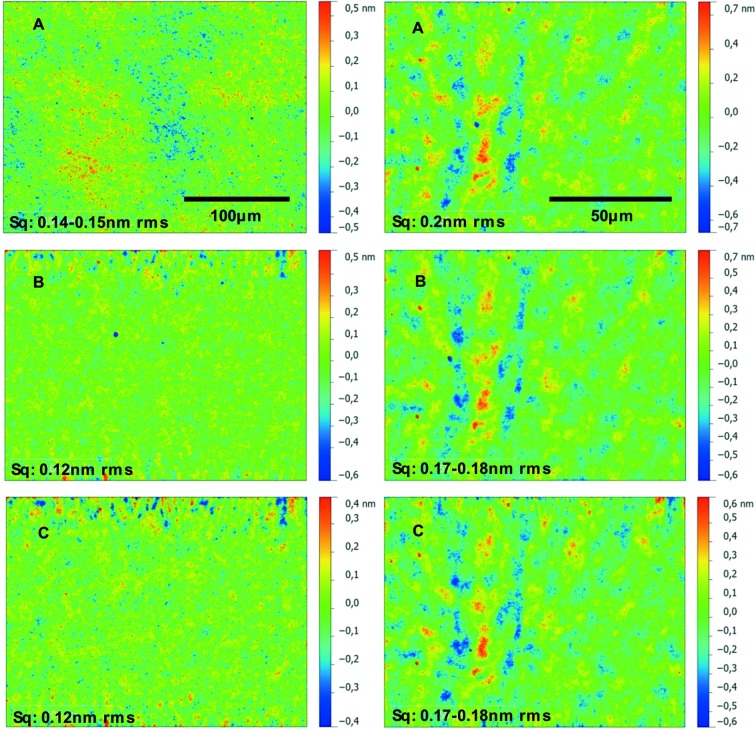
Micro-roughness values of an uncoated super-polished silicon substrate (*A*) and two Pt-coatings with a thickness of 32 nm (*B*) and 102 nm (*C*). The values were measured at three positions on each sample using white-light interferometry with magnifications of 20× (left) and 50× (right).

Micro-roughness measurements for two Pt layers with thicknesses of 32 nm and 102 nm are shown in Fig. 6 ▸ and listed in Table 1 ▸. These thickness values have been chosen because they lie in the typical range required for X-ray mirrors. The thicker value has been proposed for the MID beamline at the European XFEL. Uncoated areas have also been analysed in order to determine their surface roughness. The micro-roughness of the two Pt-coated samples was measured using a white-light interferometer (Wyant, 2002 ▸). Magnifications of 20× and 50× were used and are considered to be standard for the technique when verifying the quality of a typical X-ray mirror in the mid- and high-spatial-frequency range. The measurements indicate no significant change of the micro-roughness in the coated state for both layer thicknesses (32 nm and 102 nm) compared with the roughness of the uncoated substrate. Thus it can be concluded that the deposition process replicates the substrate roughness. In fact, it tends to slightly improve the roughness for the measured range of spatial frequencies (see Fig. 6 ▸). It is important to note that the usage of the 50× magnification objective causes a residual systematic error, which is evident in the measurements. This dominates the topography of the experimental results for all three samples. However, it is clearly shown that a slight tendency of decreasing the micro-roughness can be realised as mentioned before.

### 3.4. Thickness uniformity of various FEL coatings   

**Figure 7:**
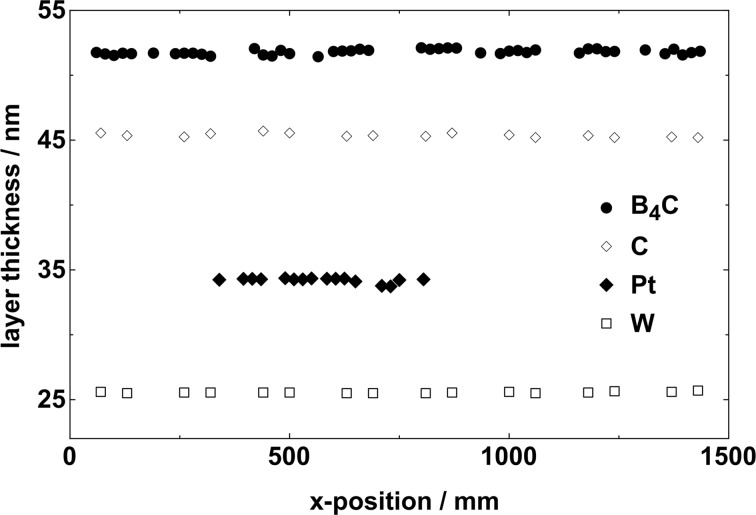
Layer thickness values of various FEL coatings such as B_4_C, C, Pt and W. The local thickness values were measured over a deposition length of 500 mm for Pt and 1500 mm for B_4_C, C and W. Regardless of the coating material, the PV value is less than 2 nm.

Fig. 7 ▸ summarizes present and previous experimental results of layer thickness values for some typical FEL coatings. The deposition length was reduced in the data for Pt, so as to reduce the cost of the target material. All data are shown as a function along the *x* direction, which is the longest direction of an X-ray mirror. It is shown that the variations in thickness values are less than 2 nm r.m.s. over the deposition length regardless of the coating material.

## 4. Outlook and conclusions   

Long FEL coatings are required at both present-day and future X-ray sources especially for the European XFEL. The technical requirements for the X-ray optics are challenging due to coherence preservation. Silicon substrates provide a very low PV shape error of less than 2 nm along an optical aperture of about 1 m length. The coating materials should retain the shape error and withstand single-shot damage. B_4_C is a very promising candidate for general use at the proposed scientific instruments. Using the HZG sputtering facility, which has a maximum deposition length of 1.5 m, numerous layers of B_4_C and Pt were manufactured and characterized. The layer thickness values were determined using X-ray reflectometry. The thickness variation achieved was 0.7 nm PV (at a mean thickness of 52 nm) along a 1.5 m length for B_4_C, which is, to our knowledge, the best value ever reported. The thickness variation achieved was 1.0 nm PV (at a mean thickness of 34.2 nm) along a 0.5 m length for platinum. The micro-roughness of the Pt layers on the silicon substrate were investigated in the mid- and high-spatial-frequency range by means of white-light interferometry. It has been found that the micro-roughness of the magnetron-sputtered Pt layers is the same as that of the substrate and independent of coating thickness in the investigated area. This indicates that a magnetron-sputtered Pt layer replicates the surface roughness of the underlying substrate. In fact, a slight improvement was found over the measured spatial frequency range.

Future activities are to coat very long (up to 1.5 m) silicon substrates for various FEL applications such as offset mirrors, distribution mirrors, benders and gratings. In the near future, ruthenium coatings will be interesting as a second stripe of a mid-*Z* element.

## Figures and Tables

**Table 1:** Micro-roughness values measured by white-light interferometry on three positions on three samples: (*A*) uncoated super-polished silicon substrate, (*B*) 32 nm-thick and (*C*) 102 nm-thick Pt layer coated on super-polished silicon substrates using magnetron sputtering The six images are shown in Fig. 6 ▸. Only one out of the three values is mentioned because all the values are the same.

		Magnification 20×	Magnification 50×
	Sample material	Micro-roughness (nm r.m.s.)	Micro-roughness (nm r.m.s.)
*A*	Uncoated Si	0.14–0.15	0.20
*B*	32 nm Pt/Si	0.12	0.17–0.18
*C*	102 nm Pt/Si	0.12	0.17–0.18
